# Quality of life in older adults according to race/color: a cross-sectional study

**DOI:** 10.1590/1516-3180.2021.0720.R1.29042022

**Published:** 2022-08-12

**Authors:** Darlene Mara dos Santos Tavares, Nayara Gomes Nunes Oliveira, Keila Cristianne Trindade da Cruz, Alisson Fernandes Bolina

**Affiliations:** I PhD. Nurse and Associate Professor, Department of Nursing Education, Postgraduate Program in Community Health Nursing, Universidade Federal do Triângulo Mineiro (UFTM), Uberaba (MG), Brazil.; II PhD. Nurse and Adjunct Professor, Department of Nursing Education, Undergraduate Program in Community Health Nursing, Universidade Federal do Triângulo Mineiro (UFTM), Uberaba (MG), Brazil.; III PhD. Nurse and Adjunct Professor, Department of Nursing, Faculty of Health Sciences, Universidade de Brasília (UnB), Brasília (DF), Brazil.; IV PhD. Nurse and Adjunct Professor, Department of Nursing, Faculty of Health Sciences, Universidade de Brasília (UnB), Brasília (DF), Brazil.

**Keywords:** Aged, Quality of life, Models statistical, Race or ethnic group distribution, Older adults, Quality of life among community-dwelling older people

## Abstract

**BACKGROUND:**

Increased longevity is accompanied by new social and health demands, such as the race/color social construct, indicating the need to identify the specific needs of older adults to maintain and improve their quality of life.

**OBJECTIVE:**

We aimed to verify the direct and indirect associations of demographic, economic, and biopsychosocial characteristics with self-assessed quality of life in older adults according to race/color.

**DESIGN AND SETTING:**

This cross-sectional study included 941 older adults living in the urban area of a health microregion in Minas Gerais, Brazil.

**METHODS:**

Older adults were divided into three groups: white (n = 585), brown (n = 238), and black (n = 102) race/color. Descriptive and trajectory analyses were performed (P < 0.05).

**RESULTS:**

Among the three groups, worse self-assessed quality of life was directly associated with lower social support scores and greater numbers of depressive symptoms. Worse self-assessed quality of life was also directly associated with a higher number of functional disabilities in basic activities of daily living and the absence of a partner among older adults of brown and black race/color. Lower monthly income and higher numbers of morbidities and compromised components of the frailty phenotype were observed among participants of white race/color, as well as lower levels of education in the brown race/color group.

**CONCLUSION:**

Factors associated with poorer self-assessed quality of life among older adults in the study community differed according to race/color.

## INTRODUCTION

Increased longevity is accompanied by new social and health demands, indicating a requirement to meet the specific needs of older populations in order to maintain and improve their quality of life (QoL).^1–4^

QoL is defined as “the individual’s perception of their position in life in relation to the context and value systems in which they are inserted, as well as their goals, expectations, standards, and concerns.”^5^ This definition highlights the complexity of the relationships among components, such as physical and psychological health, level of functional independence, social support, and interaction with the environment.^6^

Several factors can negatively influence the QoL of older adults, including the presence of comorbidities and depressive symptoms,^1^ decreased social support,^2,3^ poor physical performance,^2^ and frailty,^7^ denoting the need to maintain health in these biopsychosocial aspects.^1,2^ In an international survey of older adults, sociodemographic characteristics (female sex and low levels of education) were the main factors associated with worse QoL.^4^ In addition, a national investigation showed better QoL in white older adults compared to brown and black older adults regarding sociodemographic indicators and health conditions.^8^

Race/color is a social construct,^9^ expressed by self-reported skin color; this concept was adopted by the Brazilian Institute of Geography and Statistics when conducting the main demographic censuses.^10^ The presence of racial inequalities among older adults suggests that race/color is a marker of social position,^9^ which also reflects the distinct distribution of risk, protection, and health hazards that accumulate throughout life.^8^

The use of racial lines in epidemiological studies contributes to the analysis of the social implications of race/color on the health of different population groups.^8,9,11^ However, despite investigations identifying the differences in health and social conditions among older adults according to race/color,^4,8,9^ it is questionable whether worse self-assessed QoL can be directly attributed to racial issues and demographic, economic, and biopsychosocial factors.

Furthermore, no previous studies have assessed the relationships among these variables in older adults considering race/color and have only used models previously tested in mediation analysis. Given the complexity of these relationships, analyses that consider direct and indirect effects, such as structural equation modeling, are required for simultaneous analysis of the dependence relationship and interrelation of multiple variables, as well as estimated the direct effects mediated by other factors integrated in the causal network of the outcomes of interest.^11,12^

## OBJECTIVE

The objective of this study was to verify the direct and indirect associations of the demographic, economic, and biopsychosocial characteristics with self-assessed QoL in older adults according to race/color.

## METHODS

### Design

This cross-sectional and analytical study was guided by the Strengthening the Reporting of Observational Studies in Epidemiology^13^ Statement and conducted in a developed urban area of a health microregion in Minas Gerais (MG), Brazil.

### Sample

A multiple-stage cluster sampling technique was used for population selection. The sample size calculation considered the coefficient of determination as R^2^= 0.02 in a multiple linear regression model with 12 predictors, a significance level or type I error of α = 0.05, and a type II error of beta (β) of 0.2, resulting in a priori statistical power of 80%. Using the Power Analysis and Sample Size application (version 13; NCSS, LLC, Kaysville, Utah, United States) introducing the values described above, a sample size of at least 798 older adults was obtained. Considering a sample loss of 20%, the final number of interview attempts was 958.

Older adults aged 60 years or older living in the urban area of a health microregion in Minas Gerais, Brazil were included in the study. We excluded institutionalized older adults and older adults with communication problems such as those associated with deafness not corrected by devices, severe speech disorders, and cognitive decline, as assessed by the Mini Mental State Exam.^14^

Nine hundred and fifty-six older adults were interviewed; 31 were excluded because they presented with cognitive decline. Thus, 925 older adults were ultimately included in this study.

### Data collection

The interviews took place in the participants’ homes from March 2017 to June 2018.

### Explanatory and adjustment variables

Self-assessed QoL was measured using the question, “How would you rate your quality of life?” in accordance with the World Health Organization Quality of Life, Short Form (WHOQOL-BREF) questionnaire.^6^

Demographic data and morbidities were obtained using a structured questionnaire developed by the researchers of this study based on the literature.

Regarding functional capacity, the activities of daily living (ADL) were evaluated as follows: basic (basic ADL),^15^ instrumental (IADL),^16^ and advanced (AADL).^17^ Basic ADL were measured using the Katz Index of Independence in Activities of Daily Living,^15^ and the Lawton and Brody Instrumental Activities of Daily Living Scale^16^ for IADL. AADL were verified using the Scale of Advanced Activities of Daily Living, which includes 13 questions of a social nature.^17^ The activity performance in each of these scales was considered, with higher scores for basic ADL and lower scores for IADL and AADL indicating greater functional disability.

To verify depressive symptoms, we used the abbreviated Geriatric Depression Scale, which is comprised of 15 questions, with a total score ranging from 0–15 points.^18^

Frailty syndrome was identified through the five components of the frailty phenotype: 1) unintentional weight loss, 2) self-reported exhaustion or fatigue, 3) decreased muscle strength, 4) slow walking speed, and 5) low level of physical activity,^19^ as described in a previous study.^20^

The Brazilian version of the Short Physical Performance Battery was used to measure physical performance,^21^ with a higher score indicating better physical performance.

Social support was measured using the Social Support Scale, with the final score ranging from 20–100 points; the higher the score, the better the level of social support.^22^

### Data analysis

We built an electronic database with double entries using Excel (Microsoft Corp., Redmond, Washington, United States). The analysis was performed using Statistical Package for Social Sciences (version 24; IBM Corp., Armonk, New York, United States), and Analysis of Moment Structures software (version 24; IBM Corp.).

**Figure 1.**
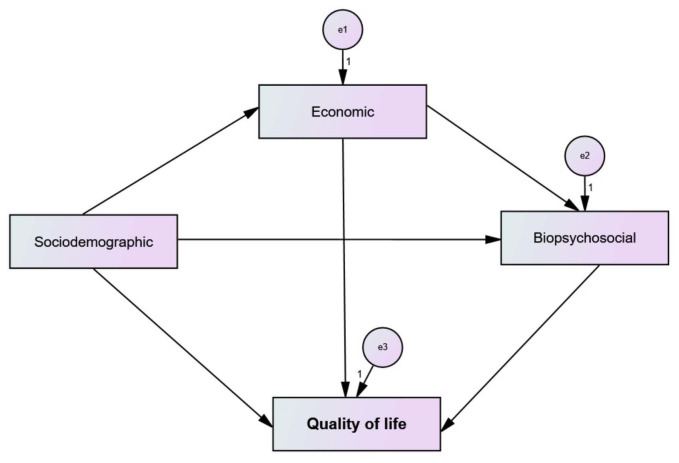
Hypothetical model.

The data were subjected to descriptive analysis. We considered that demographic, economic, and biopsychosocial characteristics were associated with self-assessed QoL through direct and indirect trajectories and developed a hypothetical model (Figure 1), which was tested through trajectory analysis^12^ and composed of observed variables, represented by rectangles and classified as endogenous and exogenous. Endogenous variables are indicated by directional arrows and measurement errors are specified by “e” in the models.^12^

1 = individual reliability; e1= measurement error 1; e2 = measurement error 2; e3 = measurement error 3.

The parameters were estimated using the asymptotic distribution-free method, and the adjustment qualities of the models were assessed according to the chi-square test (χ²) with P > 0.05; goodness of fit index ≥ 0.95; comparative fit index ≥ 0.95; Tucker-Lewis index ≥ 0.90; and root mean error of approximation ≤ 0.05.^12^ First, we tested the hypothetical model, and then re-specifications were conducted. For this purpose, non-significant pathways (P ≥ 0.05) were eliminated and modification indices (≥ 11) were calculated.^12^

In the trajectory analyses, age, education level, morbidities, functional capacity, depressive symptoms, frailty, physical performance, and social support were used in quantitative forms, including complete years of life, years of completed studies, number of morbidities, basic ADL/IADL/AADL scores, number of depressive symptoms, number of compromised components of the frailty phenotype, and physical performance and total social support scores.

In the analyzed model, the direct effects were presented through estimates of the standardized coefficients of the trajectories among the demographic, economic, and biopsychosocial variables and the self-assessed QoL. Furthermore, indirect effects were determined from intermediate trajectories among the aforementioned variables. For all tests, the type I error was set at 5% (P value < 0.05).

### Ethical considerations

This study was approved on May 9, 2017 by the Research Ethics Committee of Universidade Federal do Triângulo Mineiro (protocol number 2, 053, 520).

## RESULTS

The majority of participants were self-declared white (63.2%, n = 585), followed by brown (25.7%, n = 238), and black (11.1%, n = 102) race/color. Among the three groups, the highest percentages were observed in older adult women, aged 70–80 years, with an individual monthly income of 1 minimum wage, unmarried, and a self-rated QoL of good (Table 1).

**Table 1.** Distribution of demographic and economic characteristics and self-assessed quality of life according to race/color of older people in a health microregion of Minas Gerais, Brazil in 2018

Variables	Race/color					
	White		Brown		Black	
	n	%	n	%	n	%
**Sex**						
Female	403	68.9	157	66.0	61	59.8
Male	182	31.1	81	34.0	41	40.2
**Age group**						
60–70 years	209	35.7	96	40.3	39	38.2
70–80 years	239	40.9	98	41.2	49	48.0
80 years or older	137	23.4	44	18.5	14	13.8
**Individual monthly income, in minimum wages**						
No income	30	5.1	13	5.5	6	5.9
< 1	11	1.9	11	4.6	3	2.9
1	280	47.9	120	50.4	56	54.9
1–3	221	37.8	88	37.0	33	32.4
3–5	29	5.0	4	1.7	4	3.9
> 5	14	2.3	2	0.8	0	0.0
**Marital status**						
With partner	252	43.1	103	43.3	40	39.2
Without partner	333	56.9	135	56.7	62	60.8
**Self-assessed quality of life**						
Very bad	6	1.0	3	1.3	1	1.0
Bad	28	4.8	13	5.5	0	0.0
Neither bad nor good	134	22.9	55	23.1	18	17.7
Good	333	56.9	144	60.5	64	62.7
Very good	84	14.4	23	9.7	19	18.6

The means and standard deviations of the sociodemographic and biopsychosocial variables included in the model according to race/color in older adults in a health microregion of Brazil are shown in Table 2.

**Table 2.** Distribution of means and standard deviations of the demographic and biopsychosocial variables included in the model according to race/color of older people in a health microregion of Minas Gerais, Brazil in 2018

Variables	Race/color		
	White	Brown	Black
	Mean (±)^*^	Mean (±)^*^	Mean (±)^*^
**Age** (complete years of life)	73.9 (8.10)	72.4 (8.05)	71.6 (6.99)
**Literacy** (total years of study)	5.0 (4.20)	3.4 (3.13)	3.0 (3.01)
**Morbidities**	6.5 (3.38)	6.8 (3.46)	5.7 (3.30)
**Basic activities of daily living** (0–6 scale)	0.9 (0.34)	0.9 (0.35)	0.6 (0.23)
**Instrumental activities of daily living** (7–21 scale)	17.9 (3.16)	17.5 (3.38)	18.3 (2.81)
**Advanced activities of daily living** (0–13 scale)	5.5 (2.46)	5.0 (2.34)	5.14 (2.28)
**Depressive symptoms** (0–15 scale)	3.5 (3.33)	3.7 (3.42)	2.8 (2.60)
**Frailty** (0–5)	1.6 (1.37)	1.4 (1.23)	1.5 (1.50)
**Physical performance** (0–12 scale)	8.0 (3.24)	8.3 (3.05)	8.2 (3.27)
**Social support** (20–100 scale)	87.9 (16.87)	87.8 (17.3)	90.2 (14.68)

* ± standard deviation.

**Figure 2.**
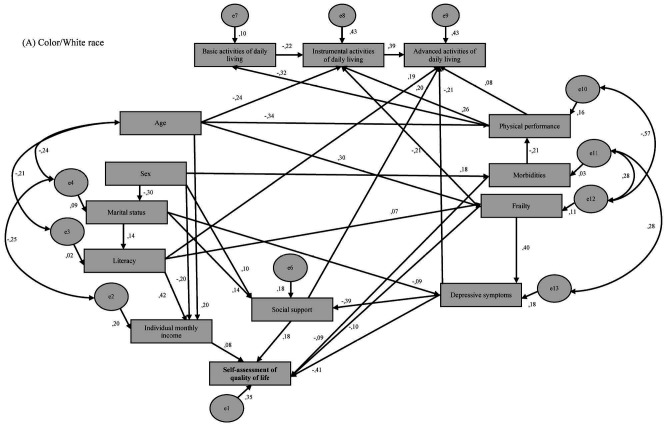

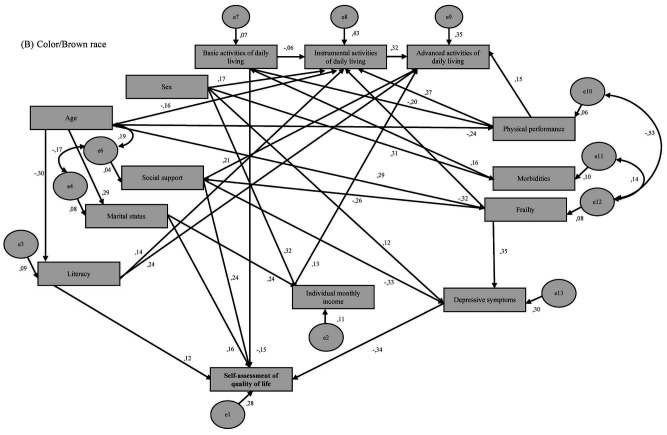

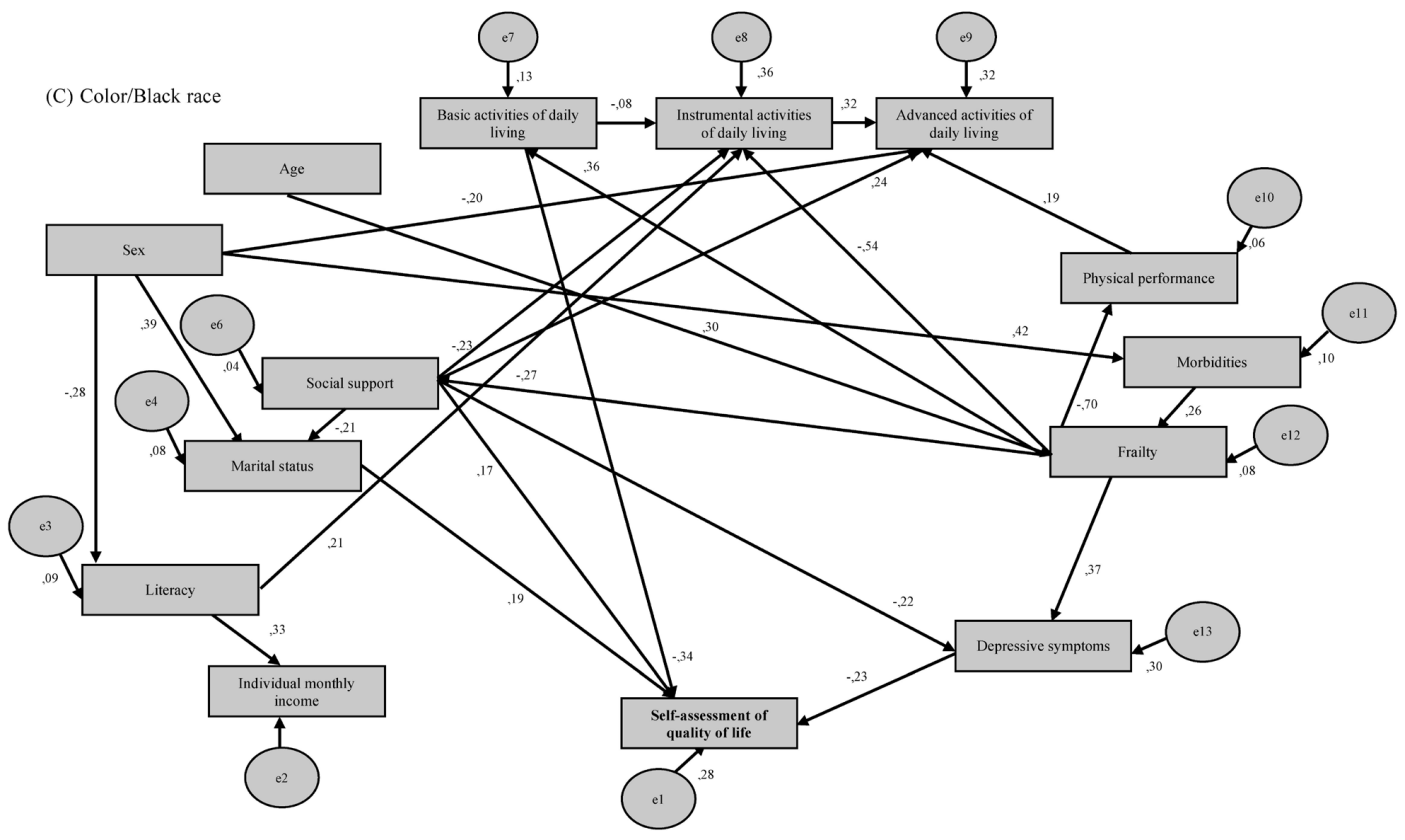
Models for analyzing the associations between demographic, economic, and biopsychosocial variables with self-assessed quality of life according to race/color of older people in a health microregion of Minas Gerais, Brazil.

Figure 2 shows the associations of demographic, economic, and biopsychosocial variables with the self-assessed QoL according to race/color in older adults in a health microregion of Brazil: white, brown, and black race/color.

(A) (χ2 (gl = 55) = 98.6; P < 0.000; comparative fit index = 0.98; goodness of fit index = 0.98; Tucker-Lewis index = 0.97; root mean error of approximation = 0.036); (B) χ2 (gl = 56) = 96.3; P = 0.001; goodness of fit index = 0.95; goodness of fit index = 0.95; Tucker-Lewis index = 0.91; root mean error of approximation = 0.050; (C) (χ2 (gl = 66) = 72.0; P = 0.286; comparative fit index = 0.98; goodness of fit index = 0.92; Tucker-Lewis index = 0.97; root mean error of approximation = 0.030).

In the three race/color groups (white, brown, and black), we found that a lower level of social support and presence of depressive symptoms were directly associated with worse self-assessed QoL (Table 3).

**Table 3.** Direct standardized coefficients for the variables associated with self-assessed quality of life according to race/color of older people in a health microregion of Minas Gerais, Brazil in 2018

Direct effects	Race/color					
	White		Brown		Black	
	Estimator	P^*^	Estimator	P^*^	Estimator	P^*^
**Self-assessed quality of life**						
Individual monthly income	0.08	0.013	–	–	–	–
Marital status	–	–	0.16	0.005	0.19	0.026
Age	–	–	–	–	–	–
Literacy	–	–	0.12	0.031	–	–
Morbidities	-0.09	0.017	–	–	–	–
Basic activities of daily living	–	–	-0.15	0.006	-0.34	P < 0.001
Instrumental activities of daily living	–	–	–	–	–	–
Advanced activities of daily living	–	–	–	–	–	–
Depressive symptoms	-0.41	P < 0.001	-0.34	P < 0.001	-0.23	0.013
Frailty	-0.10	0.009	–	–	–	–
Physical performance	–	–	–	–	–	–
Social support	0.18	P < 0.001	0.24	P < 0.001	0.17	0.050

* P < 0.05.

Among older adults of brown and black race/color, a higher number of functional disabilities in basic ADL and absence of a partner were directly associated with worse self-assessed QoL (Table 3).

In older adults of white race/color, lower individual monthly income, higher number of morbidities, and higher number of compromised components of the frailty phenotype were directly associated with worse self-assessed QoL (Table 3). Female sex, mediated by lower individual monthly income, lower social support score, and higher number of morbidities, presented an indirect association with worse self-assessed QoL among older adults of white race/color. In this group we also found that lower education level and older age, mediated by lower monthly individual income and frailty, was indirectly associated with worse self-assessed QoL.

The absence of a partner, mediated by a lower score for social support and a higher number of depressive symptoms (β = 0.02 and β = 0.04, respectively), was indirectly associated with worse self-assessed QoL.

In the group of older adults of brown race/color, a lower education level was directly associated with worse self-assessed QoL (Table 3). Lower physical performance (β = 0.03) and higher number of morbidities (β = -0.02), mediated by functional disability in basic ADL, were indirectly associated with worse self-assessed QoL. Older age mediated by education level (β = -0.04), as well as younger age mediated by the absence of a partner (β = 0.05), was associated with worse self-assessed QoL. A higher number of depressive symptoms and lower social support score mediated the association between a higher number of compromised components of the frailty phenotype and worse self-assessed QoL (β = -0.12 and β = -0.06, respectively). Female sex, mediated by a higher number of depressive symptoms (β = 0.04), was indirectly associated with worse self-assessed QoL.

Among older adults of black race/color, a greater number of compromised components of the frailty phenotype, mediated by a greater number of depressive symptoms, functional incapacity in basic ADL, and lower social support scores (β = -0.08; β = -0.12; and β = -0.04, respectively), were indirectly associated with worse self-assessed QoL. Female sex, mediated by the absence of a partner (β = 0.07), was indirectly associated with worse self-assessed of QoL.

## DISCUSSION

In this study, regardless of race/color, decreased social support was directly associated with worse self-assessed QoL and mediated by most indirect associations. In line with these results, a survey of Brazilian older adults revealed that the best self-assessed QoL was observed among older adults who met friends frequently and received social, material and emotional support from spouses, other relatives, and first-generation descendants.^2^ An investigation of older adults in Turkey found that the greater the social support, the better the self-assessed QoL.^3^ However, these surveys did not consider the effect of race/color on QoL. Older adults with less social support are more vulnerable to depressive symptoms, negative affect, and feelings of loneliness,^2,3,23,24^ which are associated with increased morbidity and mortality and worsening of self-assessed QoL.^23,24^

In the current study, we found that less social support and a higher number of depressive symptoms were also associated with worse self-assessed QoL among the three groups, similar to previous studies^1,25,26,27,28^ conducted in the same community; however, these studies did not consider race/color. These results emphasize the importance of monitoring older adults through screenings for depressive symptoms to prevent the development of the disease and adverse QoL outcomes. These findings also highlight the relevance of actions aimed at the mental health of this population, with the objective of preventing and minimizing depressive symptoms through therapeutic groups, psychological monitoring, and encouragement to practice physical exercise and participate in social activities.^29^

The association between the greater number of functional incapacities for basic ADL and worse self-assessed QoL among brown and black older adults observed in this study is partially consistent with that observed in a study conducted in the Northeast with a predominance of people of brown and black race/color.^30^ The authors assessed QoL in older adults and found that older adults of black race/color had worse QoL values compared with whites and mixed race when assessing the physical domain of QoL.^31^

The absence of a partner was another factor that negatively affected the QoL of older adults of brown and black race/color in the current study, similar to the results of another survey.^8^ The authors justified that single older people of black race/color had worse levels of social support in terms of frequency and diversity of contacts and daily instrumental support, as they essentially live alone and avoid inviting people to their homes or going out to public places^8^, which favors a worse QoL.

Other surveys conducted of older adults, most of whom were white, found an association between comorbidity,^32^ frailty,^33^ and lower income and worse QoL,^34^ which in part support the findings of the current study. Moreover, female sex was negatively related to self-assessed QoL, as also evidenced in research conducted in Uberaba (MG), with a sample of mostly white older adults.^34^ This association was notably mediated by lower monthly individual income, lower social support score, and higher number of morbidities in the current investigation. A previous study conducted on more than 90% non-Hispanic white^35^ and Japanese older adult women^36^ verified a relationship between morbidity, social support, and monthly individual income with QoL. Considering that older adult women perceive old age more negatively than men,^7^ it is possible that older adult women with morbidities, impaired social support, and socioeconomic disadvantages have greater difficulty accepting the changes that occur with the human aging process, thereby negatively impacting QoL.

Lower education level and older age, mediated by lower monthly individual income and frailty, were indirectly associated with worse self-assessed QoL among older white adults. In one study, age and education, mediated by health conditions and functional incapacity, were found to be related to QoL in older adults.^37^ Another national survey found that age equal to or greater than 80 years and education level of 4–7 and 8 or more years were negatively associated with QoL.^2^ However, these surveys were conducted in the general older population and did not assess the effect of race/color on QoL.

The absence of a partner was associated with lower QoL among older Chinese^38^ and Korean adults;^39^ which partially corroborates the findings of the present study that identified this association among people of white race/color. Older people who live without a partner are at greater risk for depression and less social support^40^ which is reflected in the self-assessed QoL, as observed in this study.

Among brown older adults, lower education levels were associated with worse self-assessed QoL, which is partially corroborated by international studies.^40,41^ In a nationwide survey, sociodemographic variables, including low education level, better determined the health status of older adults than race/color.^11^ Education level is considered a determinant factor in reducing racial differences in proactive health behaviors,^42^ which is reflected positively in the QoL of this group.

In line with the results of this study, some researchers showed that black race/color (brown and black) is associated with higher risks for comorbidities and physical decline, which are related to lower QoL.^43^ Notably, this association was mediated by functional incapacity in basic ADL, and reinforces the assumption that autonomy and independence during the human aging process are determining factors for physical, emotional, and mental health,^40^ particularly among older women of brown race/color.

Several studies have also verified advanced age as a predictor of worse QoL in the physical and mental domains of older adult African Americans with breast cancer,^44^ which may be related to higher levels of perceived discrimination throughout life.^41^ Furthermore, brown older adults generally have lower levels of education than white older adults, which is considered a main aspect of health inequity in Brazil.^8^ Therefore, the structural social inequalities present in Brazil, including disparities in access to education, can have a negative effect on the QoL of older adults, especially affecting adults with older age.

In contrast, younger older people assumably have a worse QoL due to a lack of coping skills or expectations that are typically acquired with old age.^45^ In the current study, younger age mediated by the absence of a partner was associated with worse self-assessed QoL in brown older adults. During the human aging process, the instrumental support of a partner is considered a predictor of better QoL,^2^ since living with a partner contributes to feelings of belonging and security, thereby reducing feelings of loneliness in older adults.^46^ Thus, the absence of a partner possibly in brown older people can negatively affect QoL, especially among younger older people.

In Brazil, non-white older people were four times more likely to develop this frailty syndrome,^47^ which supports the findings of the current study. These researchers highlighted that frailty is a topic of global public interest due to its impact on QoL in older adults, families, caregivers, and health and social assistance systems.^47^ However, this association was mediated by a greater number of depressive symptoms and lower social support scores, which indicates the importance of evaluating other risk factors that mediate the relationships with QoL in brown older adults, contributing to the inequities among races in healthcare.

Some researchers have observed that the intersection between sex and race/color influences the QoL of older adults.^41,45^ In line with the evidenced findings, a study with economically disadvantaged African Americans found that older adult women reported worse QoL in the physical component compared with men.^45^ Notably, in the current study, the association of women with worse self-assessed QoL in brown older people was mediated by the greater number of depressive symptoms, which supports the hypothesis that the intersectionality of sex and race/color reflects the double marginalization of health problems in older adults, including depression.^45^

Frailty, mediated by a greater number of depressive symptoms, functional incapacity in basic ADL, and less social support was indirectly associated with worse self-assessed QoL in older adults of black race/color. One possibility that justifies these findings is that older adults of black race/color are typically single, live alone, and are more vulnerable in relation to social support, especially at older ages.^8^ In another study, the authors found that older adults who live alone also tend to develop depression and lack social support.^41^ In addition, functional incapacity can lead to dependence in performing ADL, resulting in frailty and consequent worsening of QoL.^48,49,50^ Depressive symptoms may originate from different factors such as low income, living alone,^51^ and functional dependence.^52^ Thus, the presence of depressive symptoms, incapacity to perform one or more basic ADL, and decreased social support may negatively affect the frailty of older adults, thereby worsening their QoL.^50^

Female sex, mediated by the absence of a partner, was indirectly associated with worse self-assessed QoL in older adult women of black race/color. Low income and the absence of a partner may be related to the restriction of social protection and housing in these women,^53^ regardless of age. However, the loss of a partner has a negative impact on the lives of older adult women, which may be associated with low income and worse QoL self-assessment.^39^

This study had some limitations, such as the exclusion of older adults with cognitive decline, which may have favored a healthier sample; however, the possibility of selection bias was minimized since all eligible older adults were interviewed. In addition, for the purposes of analysis, a question regarding self-assessed QoL was used; however, a broader measure including the domains/facets of QoL associated with self-reported race/color may be useful for a more in-depth analysis of the data.

## CONCLUSION

Regardless of the race/color of older adults, a lower social support score and a higher number of depressive symptoms were directly associated with worse self-assessed QoL, as well as a higher number of functional disabilities in basic ADL and the absence of a partner, among older adults of brown/black race/color. The other direct associations differed among groups; in older adults of white race/color, a lower individual income and higher number of morbidities and impaired components of the frailty phenotype were directly associated with worse self-assessed QoL, whereas in older adults of brown race/color, this association was observed with lower levels of education.
